# Shear Stress Regulates Late EPC Differentiation via Mechanosensitive Molecule-Mediated Cytoskeletal Rearrangement

**DOI:** 10.1371/journal.pone.0067675

**Published:** 2013-07-02

**Authors:** Min Cheng, Xiumei Guan, Hong Li, Xiaodong Cui, Xiaoyun Zhang, Xin Li, Xu Jing, Haiyan Wu, Emil Avsar

**Affiliations:** 1 Medicine Research Center, Weifang Medical University, Weifang, Shandong, P. R. China; 2 Department of Physics, Penn State University, University Park, Pennsylvania, United States of America; Centro Cardiologico Monzino, Italy

## Abstract

**Background:**

Previous studies have demonstrated that endothelial progenitor cells (EPCs), in particular late EPCs, play important roles in endothelial maintenance and repair. Recent evidence has revealed shear stress as a key regulator for EPC differentiation. However, the underlying mechanisms regulating the shear stress–induced EPC differentiation have not been understood completely. The present study was undertaken to further investigate the effects of shear stress on the late EPC differentiation, and to elucidate the signal mechanism involved.

**Methodology/Principal Finding:**

In vitro and in vivo assays revealed that cytoskeletal remodeling was involved in the shear stress-upregulated expression of endothelial markers vWF and CD31 in late EPCs, with subsequently increased in vivo reendothelialization after arterial injury. Moreover, shear stress activated several mechanosensitive molecules including integrin β_1_, Ras, ERK1/2, paxillin and FAK, which were all involved in both cytoskeletal rearrangement and cell differentiation in response to shear stress in late EPCs.

**Conclusions/Significance:**

Shear stress is a key regulator for late EPC differentiation into endothelial cells, which is important for vascular repair, and the cytoskeletal rearrangement mediated by the activation of the cascade of integrin β_1_, Ras, ERK1/2, paxillin and FAK is crucial in this process.

## Introduction

Accelerated reendothelialization after arterial injury inhibits neointimal thickening, and is critical for the prevention and treatment of cardiovascular diseases such as atherosclerosis [1], [2]. Extensive evidence accumulated over the past years suggests that endothelial progenitor cells (EPCs) originate from bone marrow–derived progenitor cells and home to sites of vascular damage to re-establish an intact endothelial layer following the denudation of the endothelium [3]–[6]. The EPCs are heterogeneous cells that can be classified at least into early and late EPCs. Although all EPC populations have been shown to contribute to angiogenesis, only endothelial colony-forming cells (ECFCs), also termed late EPCs, have been demonstrated to possess the characteristics of a true endothelial progenitor, possesing the ability to form de novo blood vessels in vivo or become a part of the systemic circulation system [7]–[9].

Shear stress, generated by blood flow and tissue fluid flow, plays an important role in vascular tone control, development and remodeling. Impaired responses of endothelial cells to hemodynamic forces lead to the development of various vascular diseases, such as hypertension, thrombosis, aneurysm formation and arteriosclerosis [10], [11]. During the incorporation process of EPCs into tissues, the cells are exposed to shear stress. One could speculate that shear stress plays a significant role in endothelial differentiation at a later stage, simply because late EPC differentiation closely resembles the stage at which endothelial cells start to be exposed to shear stress during normal development. Previous studies, both ours and others, have shown that shear stress promotes late EPC differentiation into a mature endothelial phenotype [12], [13]. These findings suggest that late EPCs, similarly to mature endothelial cells, respond to shear stress and transmit signals into the cell, finally resulting in cell response. However, the precise mechanism by which mechanotransduction is transformed into cell response remains unclear.

Evidence shows that shear stress causes alterations in the distribution of actin filaments, resulting in their reorganization into stress fibers aligned in the direction of flow [14]. Birukov et al [15] demonstrated that acute shear stress (say for 15 min) is sufficient for cytoskeletal rearrangement, which may serve as a transducer of mechanical forces into biochemical signals into the nucleus. Furthermore, the cytoskeletal rearrangement is involved in many aspects of cellular function, such as cell movement, contraction and differentiation [16], [17]. We therefore hypothesize that the shear-induced EPC differentiation is mediated by the cytoskeletal rearrangement. To address this possibility, we evaluated the effects of shear stress on the cytoskeletal rearrangement as well as the relationship between the cytoskeletal rearrangement and the shear–induced EPC differentiation. Moreover, we also investigated the roles played by the mechanosensitive molecules including integrin β_1_, Ras, ERK1/2, paxillin and FAK.

## Results

### The Shear Stress-induced Endothelial Marker Expression was Dependent on the Cytoskeletal Rearrangement in Late EPCs

**Figure 1:**
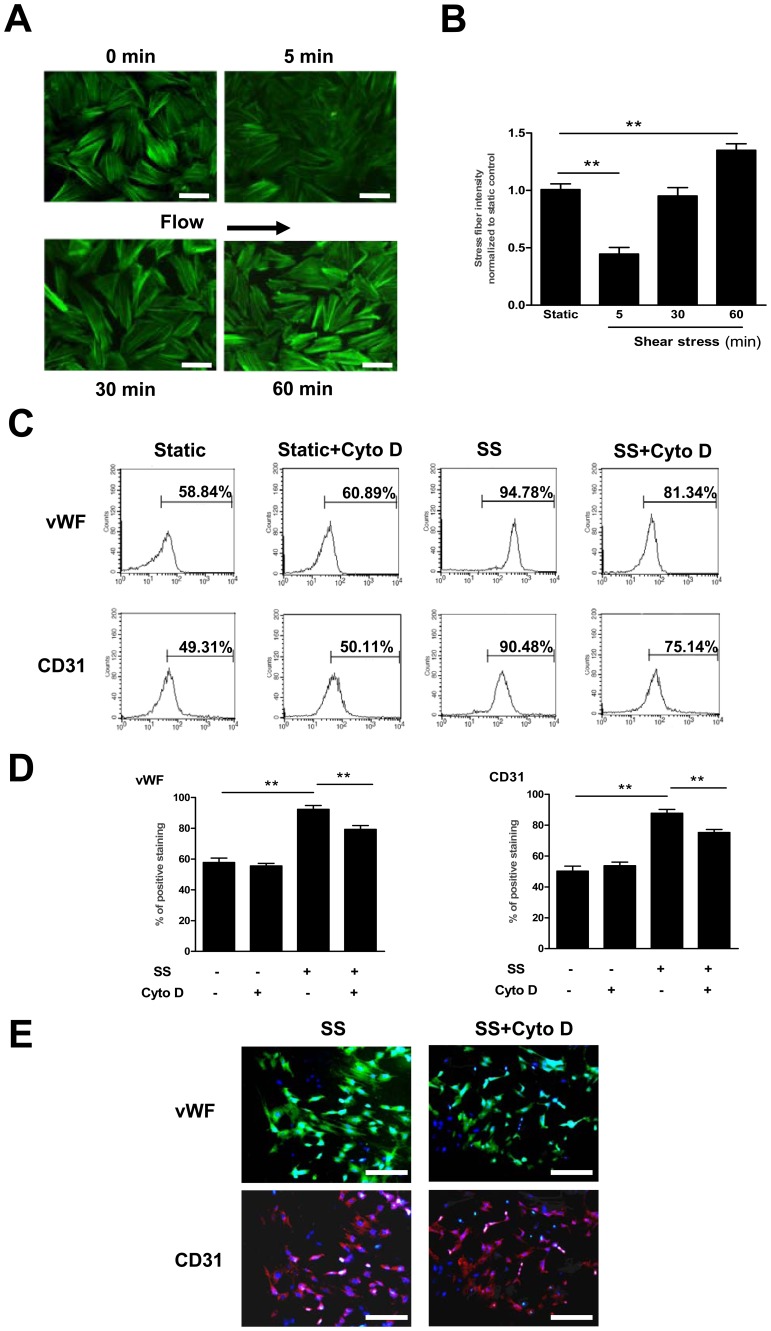
The shear stress-induced endothelial marker expression was dependent on the cytoskeletal rearrangement in late EPCs. (A) Late EPCs were kept in static condition or exposed to shear stress at 12 dyne/cm^2^ for 5, 30 or 60 min, and stained with FITC-Phalloidin to detect actin stress fibers. Bars: 100 µm. (B) Stress fibers were quantitated and normalized to the static control group. (C–D) Late EPCs were pretreated with Cyto D (1 µmol/l) for 30 min. The treated cells were then either subjected to shear stress (12 dyne/cm^2^) for 24 h, or cultured in static conditions. The protein levels of vWF and CD31 were determined by FACS. (E) Late EPCs were pretreated with Cyto D (1 µmol/l) for 30 min. The treated cells were then either subjected to shear stress (12 dyne/cm^2^) for 24 h, or cultured in static conditions. The protein expression of vWF and CD31 were determined by immunoreactivity. Bars: 200 µm. Data represent the mean±SE from three separate experiments. **(P<0.01).

Cytoskeletal rearrangement has been reported to affect the differentiation of stem cells and other signal pathways [18]. Our previous studies have demonstrated that shear stress of 12 dyne/cm^2^ induces significantly increased mRNA levels of vWF and CD31, established markers for the endothelial differentiation, at 3 h [13]. We therefore investigated whether the cytoskeletal architecture was affected by shear stress within this time frame. In late EPCs, shear stress triggered a decrease in stress fibers at 5 min, which was then followed by an increase. At 60 min the stress fibers reappeared around the periphery and were accompanied by cell alignment in the direction of flow (Figure 1 A and B).

To investigate whether the cytoskeletal rearrangement was associated with the endothelial differentiation induced by shear stress, we pretreated late EPCs with the F-actin depolymerizer Cytochalasin D (Cyto D), an inhibitor of the cytoskeletal rearrangement, before the application of shear stress (12 dyne/cm^2^, 24 h). Through FACS (Figure 1 C and D) and immunofluorescence analyses (Figure 1E), we observed that the pretreatment of EPCs with Cyto D significantly inhibited the shear stress-induced up-regulation of vWF and CD31 at the protein level. These results are consistent with our previous finding that impeding the cytoskeletal rearrangement attenuates the shear stress-induced expression of those markers at the transcriptional level [19]. Taken together, these results indicate that the shear stress-induced endothelial marker expression is dependent on the cytoskeletal rearrangement in late EPCs.

### The Shear Stress-induced Differentiation Associated with Cytoskeletal Rearrangement Enhanced the Reendothelialization Capacity in Late EPCs

**Figure 2:**
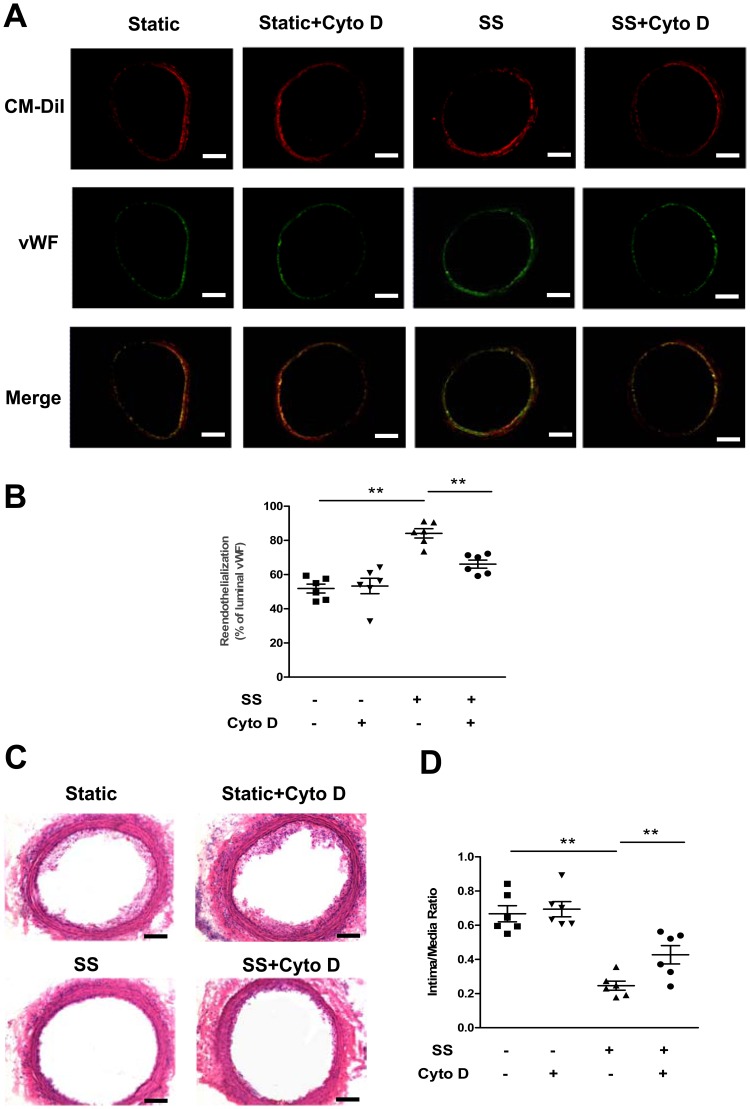
The shear stress-induced differentiation associated with cytoskeletal rearrangement enhanced the reendothelialization capacity in late EPCs. (A) Fluorescence-labeled EPCs (CM-DiI; red) were located beneath the endothelial layer, visualized by vWF immunostaining (green). Double staining with CM-DiI and vWF indicated the frequencies of EPC differentiation toward the endothelial lineage. Bars: 100 µm. (B) Quantitative analyses of reendothelialization by vWF immunofluoresence in n = 6 rats per group. (C) Vessels were perfusion-fixed 14 days after endovascular injury and EPC seeding. Representative photomicrographs of hematoxylin-eosin-stained carotid arteries. Bars: 100 µm. (D) Hematoxylin-eosin-stained cross-sections were analyzed for neointimal thickening. The intima area/media area ratios were evaluated by computer-assisted histomorphometry in n = 6 rats per group. **(P<0.01).

To investigate whether the shear stress-induced differentiation associated with cytoskeletal rearrangement contributes to the reendothelialization in vivo, late EPCs subjected to different treatments were locally infused into fresh balloon-injured carotid arteries. After 14 days, fluorescent microscopy revealed that the transplanted CM-DiI-labeled EPCs were located at the sites of injured arterial, and that the EPCs subjected to shear stress had almost formed a monolayer on the luminal surfaces (Figure 2A). As suggested by the degree of luminal vWF expression, the transplantation of shear stressed-treated EPCs significantly enhanced the reendothelialization area of denuded carotid arteries in rats (Figure 2 A and B). Moreover, a significant number of CM-DiI and vWF double positive cells were observed on the reendothelialized luminal surfaces in rats transplanted with shear stress-treated EPCs, indicating that the shear stress induced-EPC differentiation towards the endothelial lineage contributed to reendothelialization (Figure 2A). Morphometric analyses of arterial cross sections revealed a significant reduction of the neointima area in rats transplanted with shear stress-treated EPCs, in comparison to the neointima area in those transplanted with untreated-EPCs (Figure 2 C and D). However, in rats transplanted with late EPCs pre-incubated with Cyto D before the application of shear stress, a reduced number of CM-DiI and vWF double positive cells were observed on the luminal surfaces, together with an attenuated reendothelialization and increased intimal thickening (Figure 2 A, B, C and D). These results suggest that shear stress induces the late EPC differentiation by triggering cytoskeletal rearrangement, thereby leading to an enhancement of the reendothelialization in vivo.

### Integrin β_1_ was Involved in the Shear Stress-induced Cell Differentiation Associated with Cytoskeletal Rearrangement in Late EPCs

**Figure 3:**
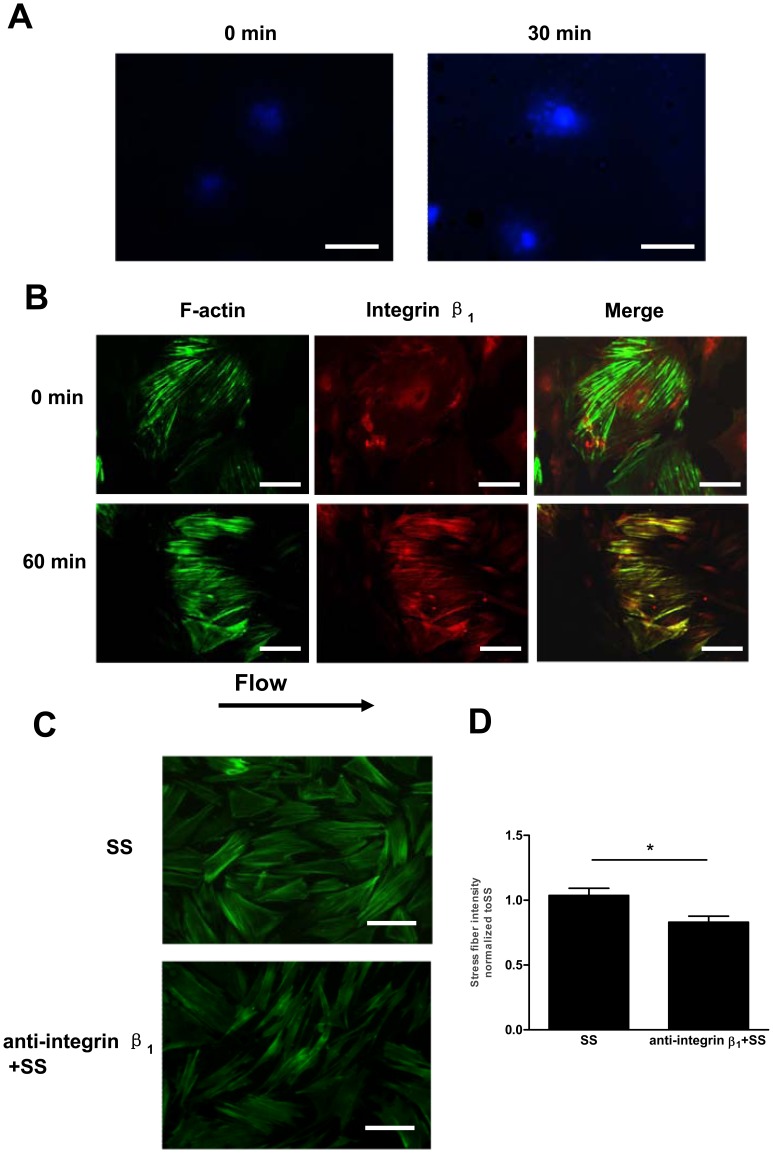
Integrin β_1_ was involved in the shear stress-induced cell differentiation associated with cytoskeletal rearrangement in late EPCs. (A) Late EPCs were exposed to shear stress (12 dyne/cm^2^) for 30 min or kept as static controls. The activated integrin β_1_ was revealed by immunostaining using HUTS-4 mAb. Bars: 100 µm. (B) Late EPCs were kept in static condition or exposed to shear stress at 12 dyne/cm^2^ for 60 min. F-actin and integrin β_1_ were stained with FITC-Phalloidin and anti-integrin β_1_, respectively. Bars: 50 µm. (C) Before being exposed to shear stress at 12 dyne/cm^2^ for 60 min, late EPCs were pretreated with anti-integrin β_1_ (50 µg/ml) for 30 min. F-actin was stained with FITC-Phalloidin. Bars: 100 µm. (D) Stress fibers were quantitated and normalized to the shear stress-treated EPCs. The results represent the mean±SE from three independent experiments. *(P<0.05).

The activation of integrins in endothelial cells by fluid shear stress has been documented to mediate cytoskeletal alignment [20]. To test whether shear stress induces the activation of integrin β_1_, late EPCs were subjected to shear stress (12 dyne/cm^2^) for 30 min [21]. The cells were then fixed and stained with HUTS-4, which selectively recognizes integrin β_1_ in its active forms. As shown in Figure 3A, the unsheared cells showed only weak HUTS-4 staining. In contrast, shear stress caused the peripheral localization of integrin β_1_, which correlated with an increased HUTS-4 staining. Immunostaining moreover revealed that, prior to fluid shear, integrin β_1_ was diffusely distributed over the cell surfaces. After the application of fluid shear for 60 min, however, integrin β_1_ became concentrated in peripheral sites and located along the stress fibers (Figure 3B).

We have previously demonstrated that the integrin subunit β_1_ plays important roles in regulating the shear stress–induced endothelial cell differentiation marker expression in late EPCs [13]. To test whether integrin β_1_ also plays a role in the shear-induced cytoskeletal rearrangement, we pretreated late EPCs with the integrin β_1_-inhibiting antibody anti-integrin β_1_ (50 µg/ml). The pretreatment of late EPCs with anti-integrin β_1_ antibody inhibited the formation of shear fibers (Figure 3 C and D), while a non-modulating integrin β_1_ antibody produced no visible effects (Data not shown).

### Ras was Essential for the Shear Stress-induced Cell Differentiation Associated with Cytoskeletal Rearrangement in Late EPCs

**Figure 4:**
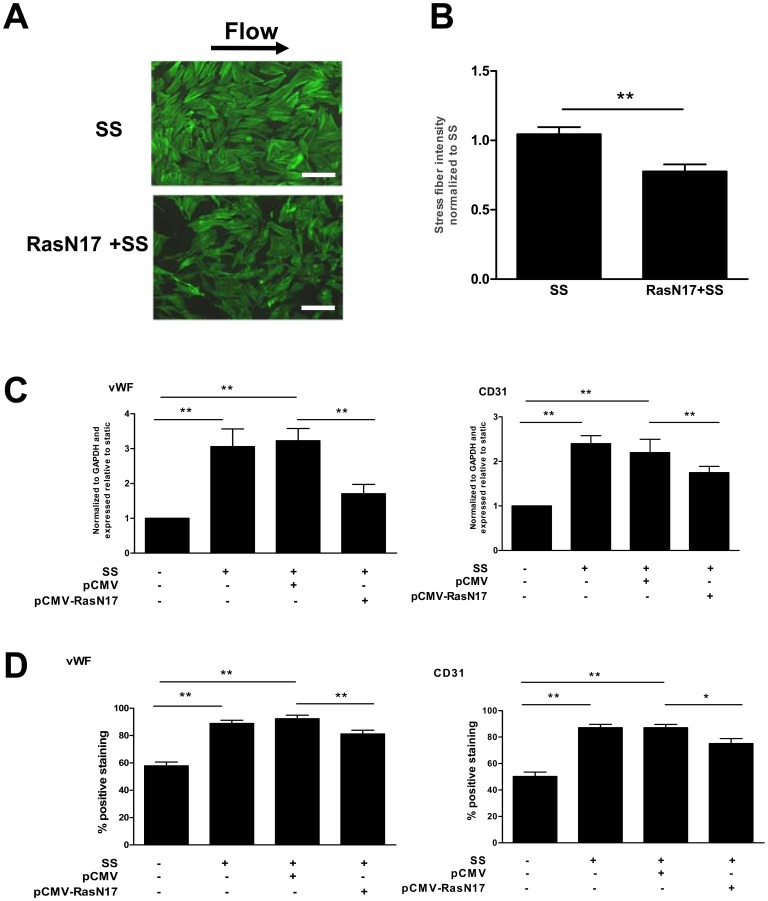
Ras was essential for the shear stress-induced cell differentiation associated with cytoskeletal rearrangement in late EPCs. (A) Late EPCs were transfected with RasN17 by the Lipofectamin 2000. The transfected late EPCs were then subjected to shear stress (12 dyne/cm^2^) for 60 min. F-actin was stained with FITC-Phalloidin. Bars: 100 µm. (B) Stress fibers were quantitated and normalized to the shear stress-treated EPCs. (C) Late EPCs were transfected either with control vector or with RasN17. The transfected late EPCs were then subjected to shear stress (12 dyne/cm^2^) for 3 h. The gene expression of vWF and CD31 was determined by real time RT-PCR. (D) Late EPCs were transfected either with control vector or with RasN17, and the transfected late EPCs were then subjected to shear stress (12 dyne/cm^2^) for 24 h, or cultured in static condition. The protein levels of vWF and CD31 were determined by FACS. The results represent the mean±SE from three independent experiments. **(P<0.01) and *(P<0.05).

There is rising evidence that the small GTPase p21ras is involved in the mechanotransduction of shear stress via the activation of integrins [22]. Our previous study showed that shear stress (12 dyne/cm^2^) triggers a rapid and transient increase in Ras activity [19]. To determine whether Ras was involved in the shear stress-induced cytoskeletal rearrangement and differentiation, the Ras-negative mutant (RasN17) was transfected into late EPCs. The late EPCs transfected with RasN17 failed to develop stress fibers (Figure 4 A and B) under shear stress, with a reduced endothelial marker expression both at the gene and protein levels (Figure 4 C and D).

### The Shear Stress-induced EPC Differentiation Associated with Cytoskeletal Rearrangement was Mediated via the Ras/ERK1/2– dependent Signal Pathway

**Figure 5:**
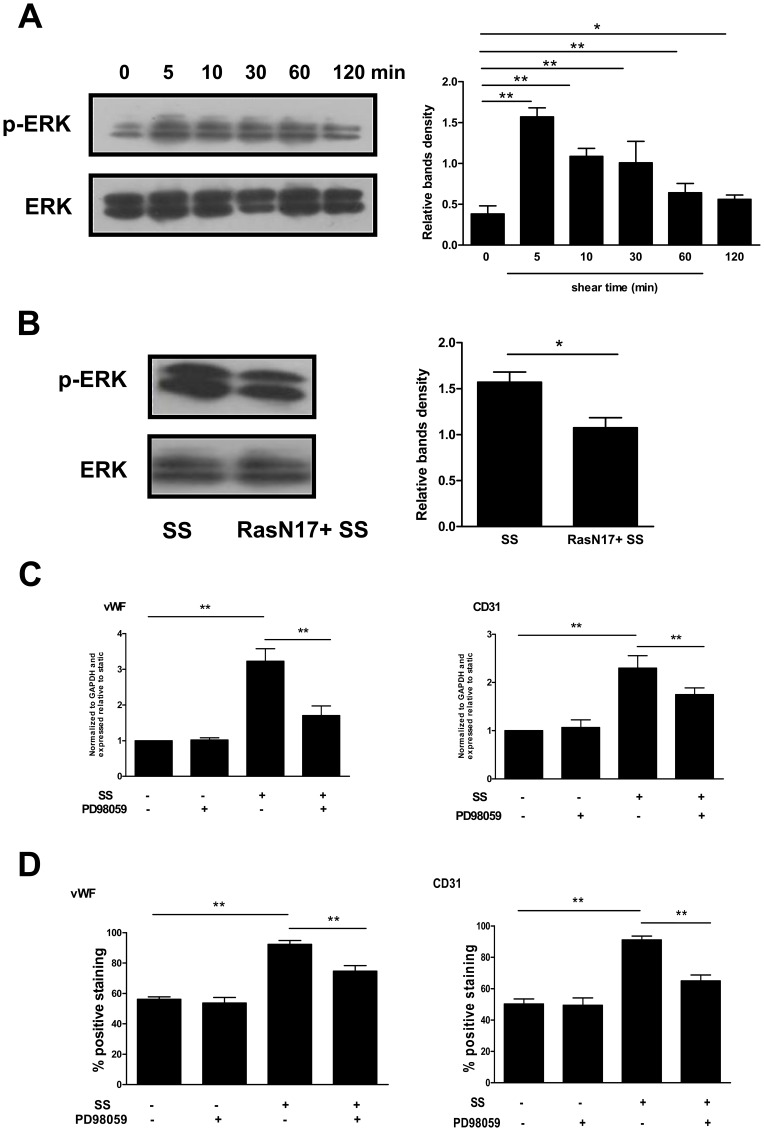
The shear stress-induced EPC differentiation associated with cytoskeletal rearrangement was mediated via the Ras/ERK1/2– dependent signal pathway. (A) Western blot was carried out with specific antibody for checking the phosphorylated ERK1/2. The total ERK1/2 served as loading control. (B) Late EPCs were transfected with RasN17. Transfected late EPCs were then subjected to shear stress (12 dyne/cm^2^) for 5 min. The activation of ERK1/2 was analyzed by Western blot. (C) Late EPCs were pretreated with PD98059 (10 µmol/l) for 30 min. The cells were then either exposed to shear stress (12 dyne/cm^2^) for 3 h, or cultured in static condition. After this, the vWF and CD31 mRNA expression was determined using real time RT-PCR. (D) Late EPCs were pretreated with PD98059 (10 µmol/l) for 30 min, and were then either exposed to shear stress (12 dyne/cm^2^) for 24 h, or cultured in static condition. The protein levels of vWF and CD31 were determined by FACS. The results represent the mean±SE from three independent experiments. **(P<0.01) and *(P<0.05).

When cells are exposed to shear stress, Ras is a mediator for ERK1/2 activation [14]. Thus, we further investigated whether the downstream of the Ras-dependent signal pathways contributed to the shear stress-induced EPC differentiation. Shear stress rapidly increased the ERK1/2 phosphorylation within 5 min which was then followed by a decline, but with the level still elevated up to 2 h (Figure 5A). As expected, the pretreatment of EPCs with RasN17 resulted in an inhibition of the ERK1/2 phosphorylation (Figure 5B). Moreover, the ERK1/2 specific inhibitor PD98059 attenuated the shear stress-induced expression of vWF and CD31 (Figure 5 C and D).

### Paxillin was Necessary for the Shear Stress-induced Differentiation Associated with Cytoskeletal Rearrangement in Late EPCs

**Figure 6:**
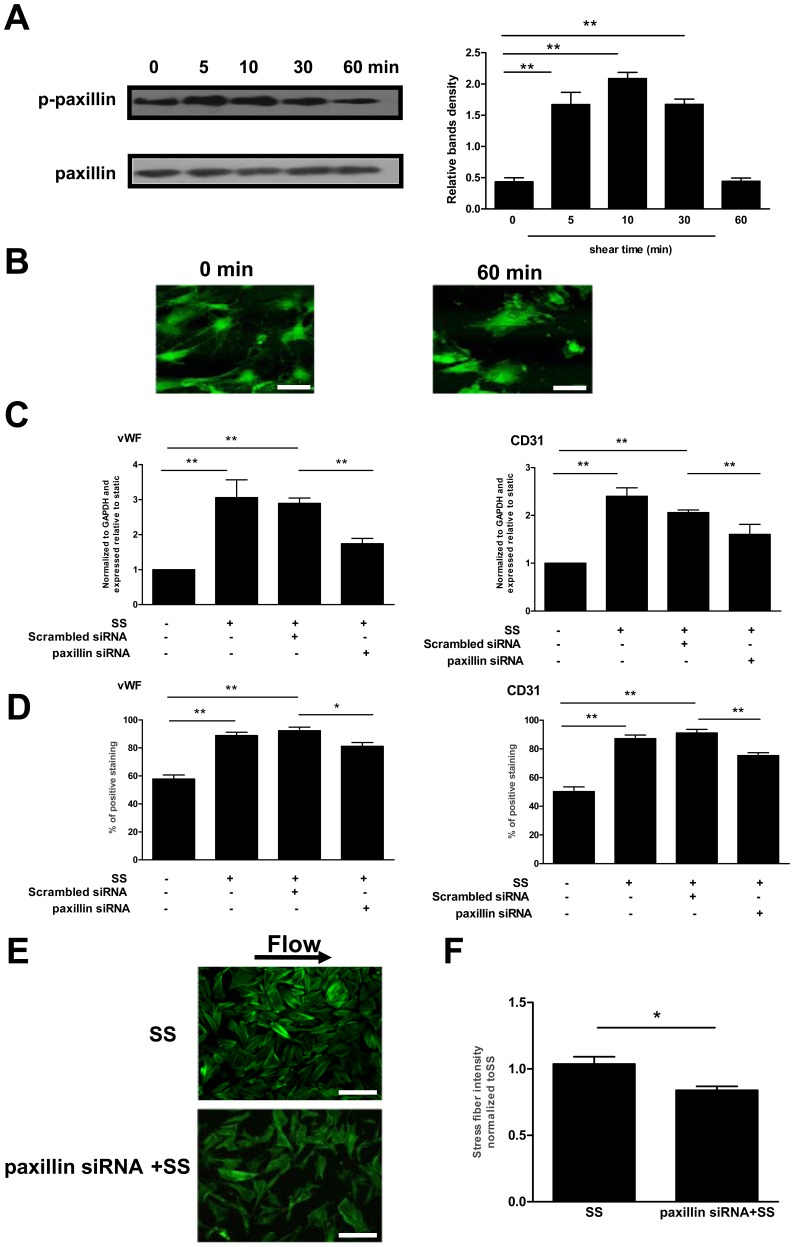
Paxillin was necessary for the shear stress-induced differentiation associated with cytoskeletal rearrangement in late EPCs. (A) Western blot was carried out with specific antibody for checking the phosphorylated paxillin. The total paxillin served as loading control. (B) Late EPCs were kept in static condition or exposed to shear stress at 12 dyne/cm^2^ for 60 min. Paxillin was stained with specific antibody. Bars: 50 µm. (C) Late EPCs were transfected either with scrambled siRNA or with paxillin siRNA by the Lipofectamin 2000. The cells were then either exposed to shear stress (12 dyne/cm^2^) for 3 h, or cultured in static condition. The gene expression of vWF and CD31 was determined by real time RT-PCR. (D) The cells were either exposed to shear stress (12 dyne/cm^2^) for 24 h, or cultured in static condition. The protein levels of vWF and CD31 were determined by FACS. (E) Late EPCs were transfected either with scrambled siRNA or paxillin siRNA by the Lipofectamin 2000. Transfected late EPCs were then subjected to shear stress (12 dyne/cm^2^) for 60 min. F-actin was stained with FITC-Phalloidin. Bars: 100 µm. (F) Stress fibers were quantitated and normalized to the shear stress treated-EPCs. The results represent the mean±SE from three independent experiments. **(P<0.01) and *(P<0.05).

Since paxillin appears to be critical for cytoskeletal remodeling [14], we evaluated its distribution and activation in shear-EPCs. When late EPCs were exposed to shear stress (12 dyne/cm^2^), phosphorylation of paxillin was increased at 5 min. The activation reached a peak at around 10 min, steadily declining afterwards (Figure 6A). Shear stress also induced the peripheral redistribution of paxillin in late EPCs (Figure 6B).

To examine the role of paxillin in the shear stress-induced cytoskeletal rearrangement and differentiation, we knocked down the paxillin expression in late EPCs by the siRNA-mediated silencing. The suppression of the paxillin expression by up to 73% using transient siRNA silencing dramatically reduced the shear stress-induced expression of vWF and CD31 in EPCs (Figure 6 C and D). When the cells were kept in static condition however, no effects were observed on the vWF and CD31 expression (data not shown). Furthermore, the shear stress-induced development of stress fibers was also blocked by paxillin siRNA (Figure 6 E and F).

### The Role of FAK in the Shear Stress-induced Cytoskeletal Rearrangement and Differentiation in Late EPCs

**Figure 7:**
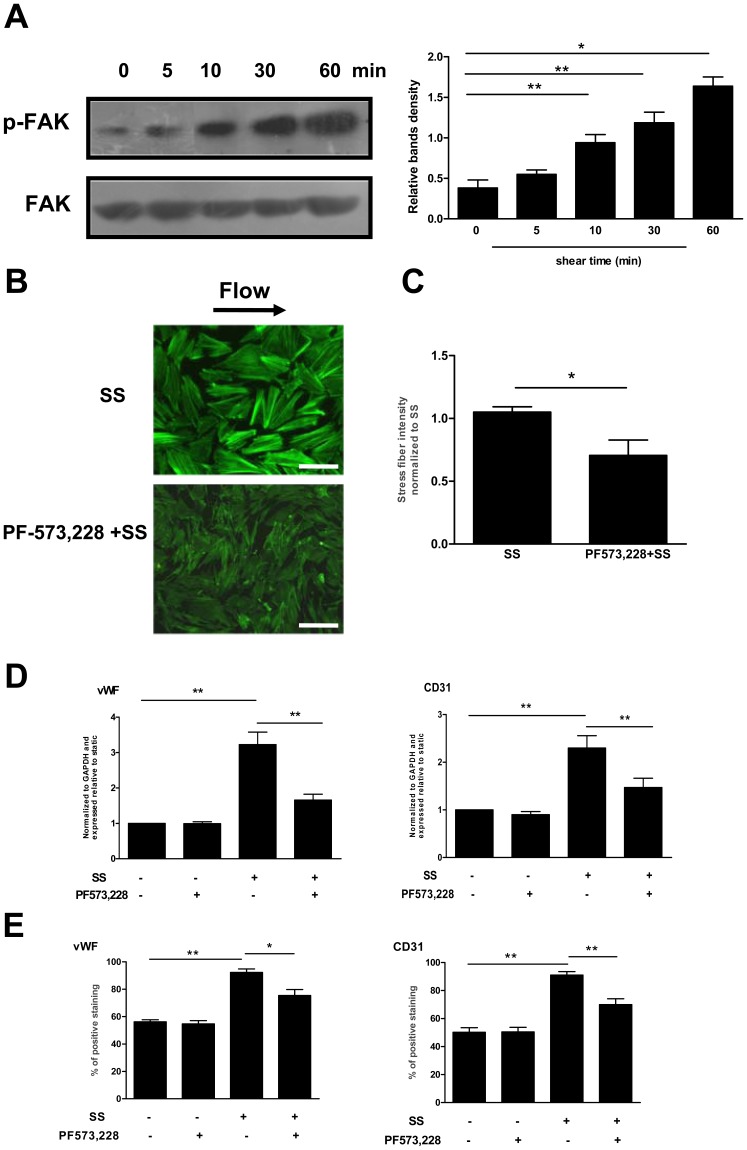
The role of FAK in the shear stress-induced cytoskeletal rearrangement and differentiation in late EPCs. (A) Western blot was carried out with specific antibody for checking the phosphorylated FAK. The total FAK served as loading control. (B) Late EPCs were pretreated with PF-573,228 (2 µmol/l) for 1 h. The cells were then either exposed to shear stress (12 dyne/cm^2^) for 60 min, or cultured in static condition. After this, F-actin was stained with FITC-Phalloidin. Bars: 100 µm. (C) Stress fibers were quantitated and normalized to the shear stress treated-EPCs. (D) Late EPCs were pretreated with PF-573,228 (2 µmol/l) for 1 h, and were then either exposed to shear stress (12 dyne/cm^2^) for 3 h, or cultured in static condition. The gene expression of vWF and CD31 was determined by real time RT-PCR. (E) Late EPCs were pretreated with PF-573,228 (2 µmol/l) for 1 h, and the cells were then either exposed to shear stress (12 dyne/cm^2^) for 24 h, or cultured in static condition for the same duration. The protein levels of vWF and CD31 were determined by FACS. The results represent the mean±SE from three independent experiments. **(P<0.01) and *(P<0.05).

Cytoskeletal remodeling induced by shear stress is regulated by interactions with focal adhesions [23]. Among several of the focal adhesion proteins, FAK is, besides paxillin, another key player in regulating the cytoskeletal organization. Thus we examined the effects of shear stress on the FAK phosphorylation. As shown in Figure 7A, shear stress (12 dyne/cm^2^) increased the FAK-397 phosphorylation in a time-dependent manner.

Moreover, treatment of late EPCs with PF-573,228, a novel small molecule inhibitor of FAK, inhibited the formation of shear fibers and the shear stress-induced EPC differentiation (Figure 7 B, C, D and E).

## Discussion

Shear stress plays a key role in endothelial function, and potentially also in the endothelial specification of stem cells or progenitor cells, for example EPCs [24], [25]. Recently, Obi et al have reported that shear stress increases the differentiation of circulating early EPCs [25]. We have previously also demonstrated that shear stress promotes late EPC differentiation in a magnitude-dependent manner, leading to significantly increased vWF and CD31 gene expression at 12 dyne/cm^2^ [13], [19].

One may ask how shear stress, as a physical agent, leads to chemical signal events in the cells. This is a fundamental cellular process that occurs at the cell–extracellular matrix contacts known as focal adhesions. At these sites, integrins are associated with the actin cytoskeleton. This interaction with actin is mediated by numerous cytoskeletal and signaling molecules [26]. Any attempt to understand the molecular basis for cellular mechanosensitivity should take into consideration the detailed structure of these sites. Our previous study has shown that shear stress increases the adhesion of late EPCs (Medical Biomechanics, accepted data). Moreover, cytoskeleton, integrin β_1_, Ras, ERK1/2, paxillin and FAK are all crucial in this process (Figure S1).

To further elucidate the possible mechanism by which the differentiation towards an endothelial lineage takes place in late EPCs, we have shown here that shear stress (12 dyne/cm^2^) leads to cytoskeletal rearrangement within 1 h, although maximal alignment requires longer times of exposure to flow (Figure S2). Cytoskeletal rearrangement may facilitate the contact and translocation of various signaling molecules, which in turn contribute to the activation of the upstream signaling molecules that regulate EPC differentiation, such as PI3K and Akt [25], [27]. Moreover, the mechanical coupling of the cytoskeleton to the nucleus enables the shear stress-induced cytoskeletal rearrangement to influence the packing of DNA in the nucleus [28], altering the endothelial cell differentiation marker gene expression. The present data show that interference with these reorganization processes using the F-actin depolymerizer Cyto D results in a decreased expression of the shear stress-induced endothelial differentiation markers in late EPCs. In parallel, in agreement with the findings of Xia et al [29], transplantation of EPCs treated with shear stress facilitated reendothelialization and reduced neointimal lesions following arterial injury, as compared to those cells kept under static conditions. This may at least partly be due to the shear stress-induced differentiation of late EPCs. Furthermore, pretreatment with Cyto D attenuated the shear stress-induced enhancement of the in vivo reendothelialization capacity of late EPCs. These results indicate that the cytoskeletal rearrangement plays an important role in the shear stress-induced differentiation of late EPCs which are involved in the EPC-mediated reendothelialization after arterial injury. Thus, further insights into the early-time mechanical processes of the cytoskeletal rearrangement will help us to better understand the mechanisms behind the shear stress-induced late EPC differentiation.

A variety of data have demonstrated that integrins function as mechanotransducers [22]. Recently, we have shown that shear stress upregulates the expression of integrin β_1_ and β_3_, resulting in late EPC differentiation [13]. Since integrin β_1_ may play the dominant role in the function of EPCs [30], it is possible that the shear stress-induced cytoskeleton changes are facilitated by integrin β_1_-related signals. In the present study, we found that integrin β_1_ was activated by shear stress, and became concentrated at peripheral sites and located along the stress fibers, facilitating the formation of focal adhesions [31]. In late EPCs pretreated with anti-integrin β_1_ the shear stress-induced cytoskeletal rearrangement was inhibited, suggesting that integrin β_1_ is necessary for transmitting mechanical signals across the membrane to the cytoskeleton.

There is rising evidence that shear stress activates integrins, which in turn activate the small GTPase p21ras [22] that is one of the earliest links between rapid mechanotransduction events and the effects of shear stress on downstream signal transduction cascades [14]. Using pulldown assays we previously showed that the shear stress-induced activation of Ras is rapid and transient [19]. This increase is dependent on integrin β_1_ related signals since the blockade of integrin β_1_ prevented the shear stress–mediated Ras activation (Figure S3). This finding suggests that integrin β_1_ determines the activation of Ras in response to shear stress. Moreover, inhibition of Ras activity through the expression of RasN17 partly abolished the shear stress-induced cytoskeletal rearrangement and EPC differentiation.

When cells are exposed to shear stress, Ras is a mediator for ERK1/2 activation [14]. We thus investigated whether ERK1/2 contributes to the shear stress-induced EPC cytoskeletal rearrangement and differentiation. Firstly, we observed that ERK1/2 was rapidly and transiently activated by shear stress. Such responses have their physiological significance. EPCs mobilized from bone marrow to peripheral blood attach to existing endothelial cells. Rapid activation of signaling molecules, such as Ras and ERK1/2 would help EPCs to adapt to the sudden onset of fluid flow. After a while, just like mature endothelial cells, EPCs need a desensitization mechanism to protect them from the continuous stimulation imposed by hemodynamic forces. Next, we found that the pretreatment of EPCs with RasN17 resulted in a partial inhibition of the ERK1/2 phosphorylation. Finally, the data in the present study demonstrate that the inhibition of ERK1/2 with PD98059 blocks the shear stress-induced upregulation of the endothelial markers. Thus, the shear stress-activated ERK1/2 is downstream of the signal-transduction cascade of Ras, and promotes late EPC differentiation possibly via the cytoskeletal rearrangement.

A growing body of evidence suggests that cytoskeletal remodeling induced by mechanical factors may also be regulated by focal adhesions [32]. Among the several focal adhesion related molecules, paxillin is a multidomain adaptor protein that integrates signals from integrins to actin cytoskeletal remodeling effectors [33], such as FAK [34]. Our experimental data have demonstrated that exposure of late EPCs to shear stress increases paxillin and FAK activation. Moreover, the inhibition of paxillin or FAK decreased the shear stress-induced cytoskeletal rearrangement and differentiation in late EPCs. These results suggest that paxillin and FAK might contribute to late EPC differentiation associated with the cytoskeletal rearrangement.

Taken together, the present study demonstrates that cytoskeletal remodeling is involved in the shear stress-upregulated expression of endothelial markers vWF and CD31 in late EPCs, with subsequently increased in vivo reendothelialization after arterial injury. Moreover, this increase was observed to be mediated by the activation of the cascade of integrin β_1_, Ras, ERK1/2, paxillin and FAK. Although further studies are needed to confirm the hierarchy and the relationship between these mechanosensitive molecules, the present results may provide new insights into the underlying mechanisms by which shear stress regulates the EPC differentiation.

## Materials and Methods

### Isolation of Bone Marrow Mononuclear Cells, Cell Culture and Identification of Late EPCs [13]

Whole bone marrow was isolated from the femurs and tibias of the Sprague-Dawley rats (150 to 175 g, Weifang Medical University, China) after culling with an overdose of anesthetic (100 mg/kg ketamin and 5 mg/kg xylazine), death being confirmed by cervical dislocation. The BM MNCs were fractionated by density gradient centrifugation (Histopaque®-1083, Sigma, USA), plated on dishes pre-coated with fibronectin (Roche, Germany), and maintained in complete EGM-2MV medium (supplemented with EGM-2 bullet kit, including 5% fetal calf serum, recombinant VEGF, recombinant bFGF, Invitrogen, USA). After 4 days in culture, unattached cells were removed with PBS, after which fresh medium was added. Three to five passages of cells, namely late EPCs, were used in the experiments.

The investigation has conformed to the Guide for the Care and Use of Laboratory Animals published by the United States National Institutes of Health (NIH Publication No. 85-23, revised 1996). All protocols involving animals were approved by the Committee on the Ethics of Animal Experiments of Weifang Medical University (Permit Number: 5876).

To identify the late EPCs, tube formation on Matrigel (Becton-Dickinson, USA) was evaluated according to the manufacturer’s instructions. The cells were further characterized by the uptake of 1,1′-dioctadecyl- 3,3,3′,3′-tetramethylindo-carbo-cyanine-labeled acetylated low density lipoprotein (Dil-acLDL, Molecular probes, USA), and by fluorescein isothiocyanate labeled Anti-Ulex Europaeus Lectin 1/UEA1 (FITC-UEA-1, Sigma, USA) staining. Cells demonstrating double-positive fluorescent were identified as differential late EPCs. Moreover, the cellular expression of von Willebrand factor (vWF, Sigma, USA, dilution 1∶200), CD31 (eBioscience, USA, dilution 1∶100), VEGFR2 (eBioscience, USA, dilution 1∶100) and VE-cadherin (BD, USA, dilution 1∶400) was analyzed by fluorescence-activated cell sorter (FACS Calibur, BD, USA). (Figure S4).

### Shear Stress Experiments

Shear stress was applied to the EPCs using a flow chamber system that has been described previously [13]. The intensity of shear stress was calculated using the following formula: 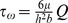, where 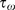 is the shear stress, 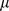 the medium viscosity (0.0077 g/cm•s), Q the volumetric flow rate (2.05 cm^3^/s), h the chamber height (0.03 cm) and b the chamber width (2.5 cm).

### Fluorescent Staining

Late EPCs were fixed with 4% paraformaldehyde in PBS for 10 min and blocked in PBS containing 1% BSA for 30 min at room temperature. vWF, CD31, integrin β_1_ or paxillin was stained with special antibodies for vWF (Sigma, USA), CD31 (eBioscience, USA), integrin β_1_ (Millipore, UAS) or paxillin (Santa Cruz Biotechnology, Inc, USA) respectively. F-actin was stained with FITC-Phalloidin (Enzo Life Sciences, USA). The images were acquired by using a fluorescence microscope (Leica, Germany).

### Stress Fiber Quantization [35]

The Image J threshold tool was used to determine an optical density limit that encapsulated most of the visible F-actin bundles, eliminating background fluorescence. The stress fiber intensities were analyzed by the ROI manager. The ratio between the mean fluorescence intensity and the cell area was calculated to account for changes in the cell cytoskeleton due to shear stress.

### RNA Isolation and Quantitative Reverse Transcription-polymerase Chain Reaction

Total cellular RNA was isolated with TRIzol reagent (Invitrogen, USA) and reverse-transcribed to cDNA using the SYBR® PrimeScript® RT-PCR Kit (Takara, Japan) at 37°C for 15 min. The gene expression was evaluated by SYBR® Premix Ex Taq™ (Takara, Japan). Rat vWF and CD31 were amplified with the specific primers. GAPDH was used as a housekeeping gene, in order to normalize the expression target gene. Primer sequences used are listed in Table S1. The thermal cycling conditions were as follows: 30 seconds at 95°C for pre-denaturation, 40 cycles for 15 seconds at 95°C for denaturation, 1 minute at 59°C for annealing, and 10 seconds at 72°C for elongation. At the end of each cycle, the fluorescence emitted by the SYBR Green I was measured. After the completion of the cycling process, samples were immediately subjected to a temperature ramp for melting curve analysis. The relative gene expression was analyzed by the 2^–ΔΔCt^ method.

### Fluorescence-activated Cell Sorter Analysis (FACS)

The protein expression of vWF and CD31 was also determined by FACS. Cells were trypsinized and incubated with antibodies for 1 h. For the detection of vWF, the cells were permeabilized with 0.1% Triton X-100 before incubation with the antibody. Typically, around 20,000 late EPCs were measured for fluorescent intensity per experiment.

### Animal Model, Cell Transplantation, Morphometric Analysis of Reendothelialization and Neointima Formation [36], [37]

Rats (300∼350 g) were anesthetized by an intraperitoneal injection of ketamin (100 mg/kg) and xylazine (5 mg/kg), and the right carotid artery was exposed. Surgery was carried out using a dissecting microscope. A Fogarty 2F embolectomy catheter (Edwards Lifesciences, Unterschleissheim, Germany) was introduced into the external carotid, advanced to the common carotid, inflated, and withdrawn three times with rotation. Late EPCs (1×10^6^) labeled with CM-Dil (Invitrogen, USA) were suspended in 150 µl PBS supplemented with 20% (v/v) rat serum and heparin (20 U/ml). The cell solution was instilled and incubated in the freshly injured arterial bed for 25∼30 min. Unbound cells were removed by rinsing the isolated arterial segment with PBS. Finally, the catheter was removed, and the blood flow was restored. On day 14, animals were euthanized and perfusion-fixed with 10% buffered formalin. Vessels were embedded in O.C.T. and frozen in liquid nitrogen. Histomorphology cross-sections were stained with hematoxylin/eosin and examined for intima to media area ratio.

Moreover, injected late EPCs were identified as CM-DiI positive cells. Endothelial cells were identified by staining with Abs against vWF. Reendothelialization was quantified by measuring the length of the vWF-positive endothelial layer as a percentage of the total luminal circumference using image analysis software (ImagePro Plus, version 4.1).

### Ras Activation Assays

Ras activation assay was measured according to the manufacturer’s instructions, by pull-down assay using the Ras binding domain of Raf1 fused to glutathione S-transferase (GST) (Thermo, USA). Briefly, cells were snap frozen and lysed in a buffer containing HEPES (25 mmol/l, pH 7.5), NaCl (250 mmol/l), 1% Nonidet P-40, MgCl_2_ (10 mmol/l), EDTA (5 mmol/l) and glycerol (10%). Lysate was then incubated with GST-Raf1-RBD (1 h, 4°C), which was subsequently recovered by centrifugation, washed twice with lysis buffer, boiled in SDS sample buffer, and subjected to 12% SDS-polyacrylamide gel electrophoresis (PAGE). Activated Ras was detected by Western blot.

### Gene Transfer

For some experiments, subconfluent cells were transfected with pCMV RasN17 (Clontech, USA) or paxillin (Sense: 5′- CGUCACUGUCAGAUUUCAATT-3′; Antisense: 5′-UUGAAAUCUGA CAGUGACGTT-3′) small-interfering RNA (50 nmol/L siRNA; Santa Cruz Biotechnology, Inc, USA) using Lipofectamin 2000 (Invitrogen) according to the manufacturer’s instructions. 48 h after the transfection, the protein expression was analyzed by Western blot.

### Western Blot Analysis

Cellular protein was extracted in 150 µl of 1×SDS loading buffer (62.5 mmol/l Tris–HCL pH 6.8, 2% SDS, 10% glycerol, 50 mmol/l DTT, 0.1% bromphenol blue) in the presence of 0.1% EDTA-free protease inhibitor cocktail, 1 mmol/l sodium orthovanadate and 1 mmol/l sodium fluoride. Protein was quantified using the bichoninic acid assay (BCA; Pierce Biotechnology, Rockford, IL) according to the manufacturer’s instructions. Equal amounts of protein (50 µg) were separated through a 12% SDS–PAGE, and transferred to a PVDF membrane. Membranes were blocked in 5% milk-TBST, followed by overnight incubation with appropriate primary antibodies, such as p-ERK1/2 (Cell signal, 1∶300 dilution), p-paxillin (Cell signal, 1∶100 dilution) or p-FAK (Cell signal, 1∶100 dilution). The total amounts of ERK1/2, paxillin and FAK served as control measures. Membranes were then washed with TBST, and incubated with secondary antibody conjugated to HRP (Santa Cruz). Immunoreactive bands were visualized by chemiluminenscence (Amersham Pharmacia ECL), and the resulting autoradiograms were analyzed by densitometry.

### Statistical Analysis

Unless otherwise indicated, results are expressed as mean ± SE. Statistical analyses were performed using Student’s t test or one-way ANOVA followed by Tukey’s test, and P<0.05 was considered statistically significant. All data were analyzed using SPSS software (version 15.0; SPSS, Chicago, IL, USA).

## Supporting Information

Figure S1 **Numerous cytoskeletal and signaling molecules were involved in the increased adhesion of late EPCs induced by shear stress.** Late EPCs were pretreated either with siRNA or the inhibitor for different cytoskeletal and signaling molecules, such as integrin β_1_, Ras, paxillin and FAK, and then sheared at 12 dyne/cm^2^ for 24 h. Late EPCs with equal cell numbers were re-seeded on fibronectin-coated culture dishes and incubated for 30 min at 37°C. After non-adherent cells were removed by washing, the adherent cells were counted independently in six random high-power (×100) microscope fields (HPF)/well by three observers unaware of the treatments. **(P<0.01) and *(P<0.05) vs. shear stress exposure only.
(TIF)

Figure S2 **Prolonged shear stress (12 h) resulted in cytoskeletal reorientation in the direction of flow in late EPCs.** Late EPCs were kept in static condition or exposed to shear stress at 12 dyne/cm^2^ for 12 h, and stained with FITC-Phalloidin to detect actin stress fibers. Bars: 100 µm.
(TIF)

Figure S3 **Blockade of the integrin β_1_ activation inhibited the shear stress–induced increase in Ras activity.** Late EPCs were pretreated with anti-integrin β_1_ (50 µg/ml) for 30 min and then sheared at 12 dyne/cm^2^ for 5 seconds. The levels of Ras-GTP were monitored via Western blot. The results represent the mean±SE from three independent experiments. *(P<0.05).
(TIF)

Figure S4 **Characterization of late EPCs derived from rat bone marrow.** (A) Late EPCs showed characteristic homogeneity and cobblestone-like morphology. Bars: 400 µm. (B) Representative images of capillary-like tubes formed on Matrigel by late EPCs. Bars: 400 µm. (C) Late EPCs were identified as double-positive for Dil-acLDL (red) uptake and lectin (green) binding affinity. Bars: 200 µm. (D) FACS analysis showing the immuno-phenotype of late EPCs using several endothelial cell-specific markers: vWF, CD31, VEGFR-2 and VE-cadherin.
(TIF)

Table S1 Primers used in Real-time RT-PCR.
(DOC)
