# Supplementary figures and images for: Shear Stress Regulates Late EPC Differentiation via Mechanosensitive Molecule-Mediated Cytoskeletal Rearrangement
Source: PLoS One. 2013 Jul 2;8(7):e67675. doi: 10.1371/journal.pone.0067675 (PMC3699607; doi:10.1371/journal.pone.0067675)

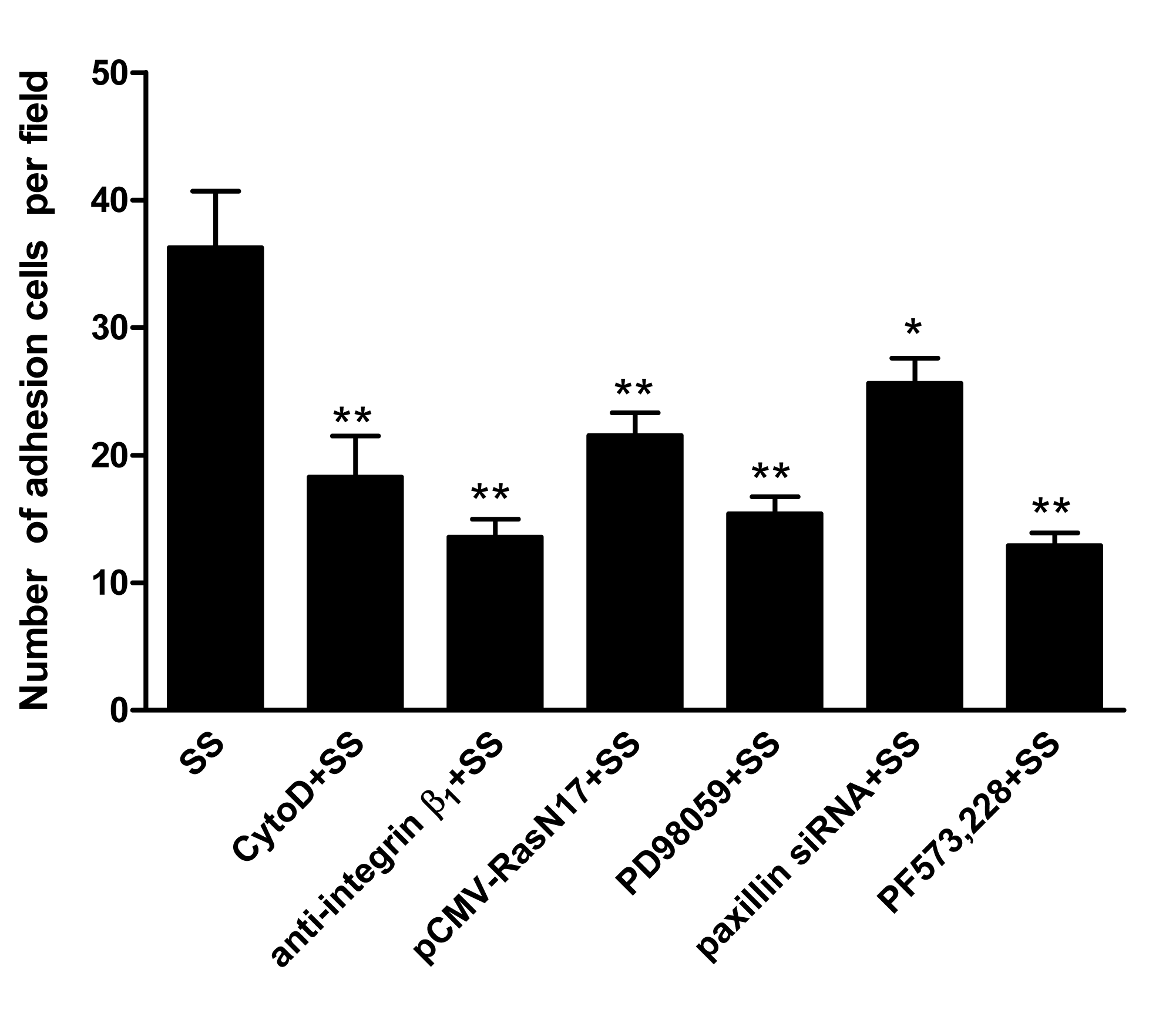

Supplement: Figure S1 — Numerous cytoskeletal and signaling molecules were involved in the increased adhesion of late EPCs induced by shear stress. Late EPCs were pretreated either with siRNA or the inhibitor for different cytoskeletal and signaling molecules, such as integrin β1, Ras, paxillin and FAK, and then sheared at 12 dyne/cm2 for 24 h. Late EPCs with equal cell numbers were re-seeded on fibronectin-coated culture dishes and incubated for 30 min at 37°C. After non-adherent cells were removed by washing, the adherent cells were counted independently in six random high-power (×100) microscope fields (HPF)/well by three observers unaware of the treatments. **(P<0.01) and *(P<0.05) vs. shear stress exposure only. (TIF) [file pone.0067675.s001.tif]

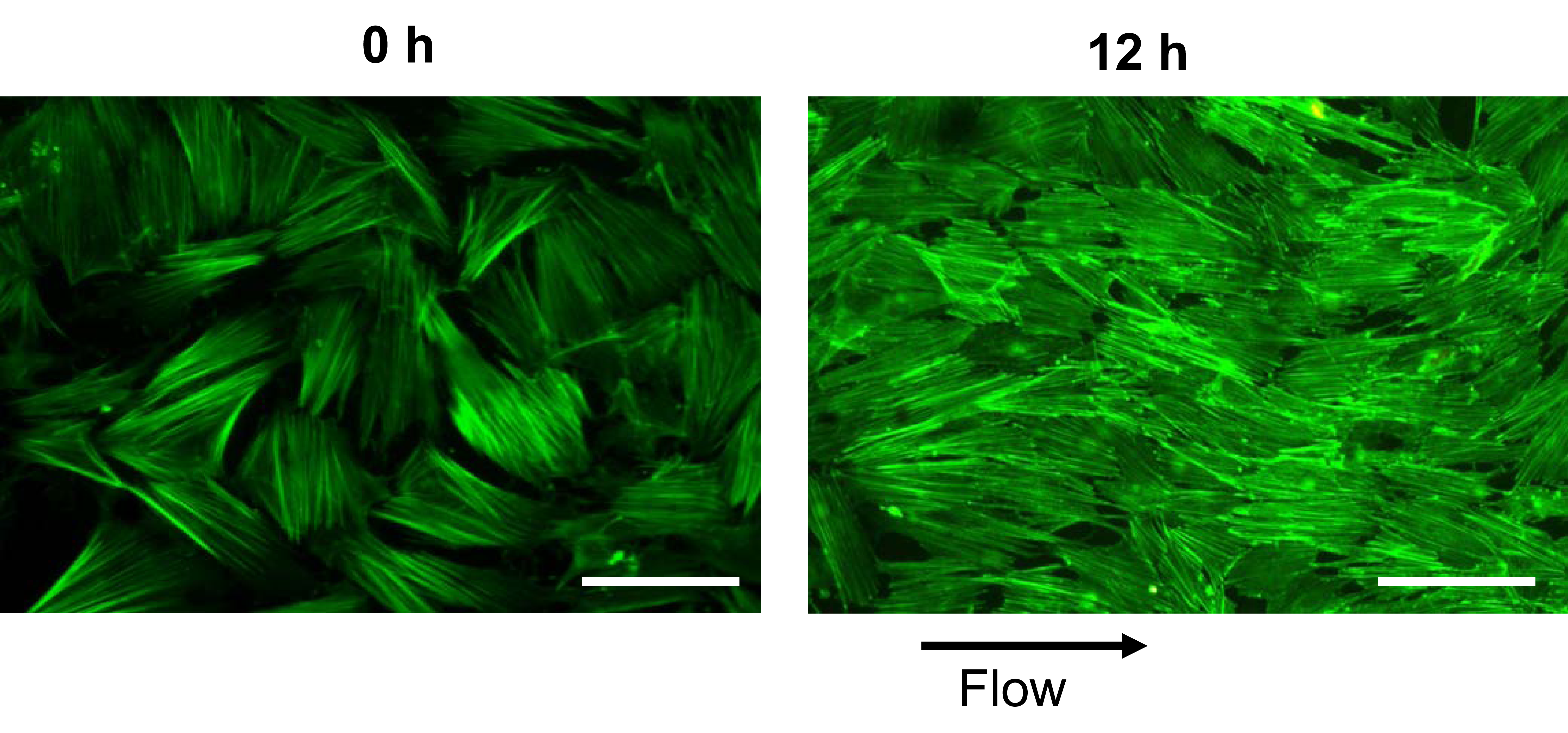

Supplement: Figure S2 — Prolonged shear stress (12 h) resulted in cytoskeletal reorientation in the direction of flow in late EPCs. Late EPCs were kept in static condition or exposed to shear stress at 12 dyne/cm2 for 12 h, and stained with FITC-Phalloidin to detect actin stress fibers. Bars: 100 µm. (TIF) [file pone.0067675.s002.tif]

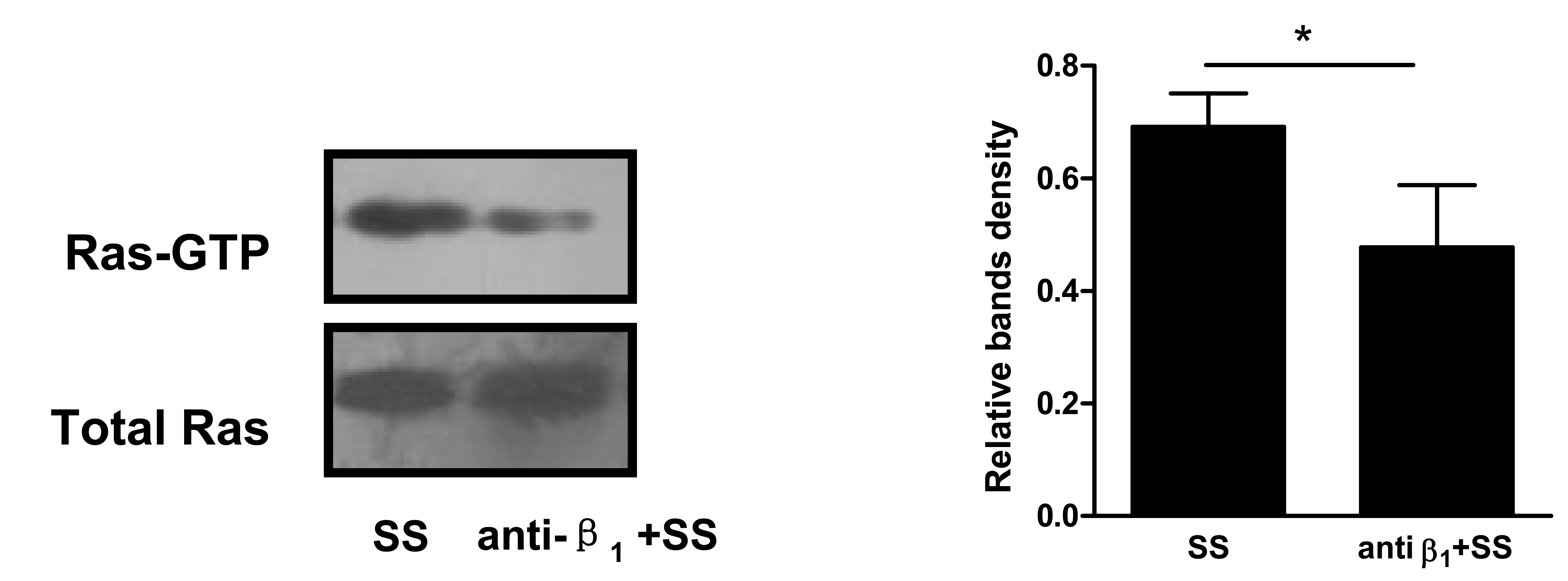

Supplement: Figure S3 — Blockade of the integrin β1 activation inhibited the shear stress–induced increase in Ras activity. Late EPCs were pretreated with anti-integrin β1 (50 µg/ml) for 30 min and then sheared at 12 dyne/cm2 for 5 seconds. The levels of Ras-GTP were monitored via Western blot. The results represent the mean±SE from three independent experiments. *(P<0.05). (TIF) [file pone.0067675.s003.tif]

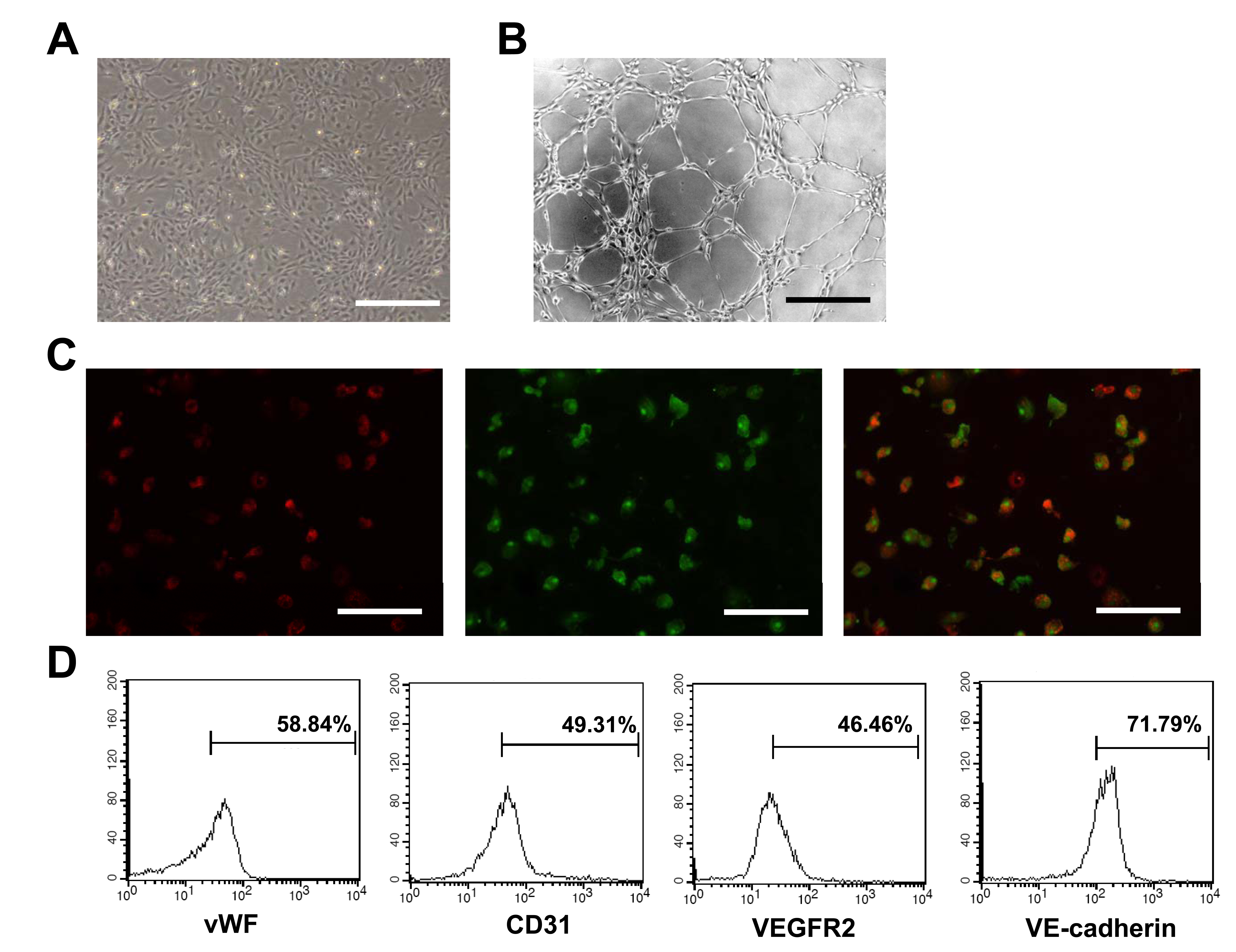

Supplement: Figure S4 — Characterization of late EPCs derived from rat bone marrow. (A) Late EPCs showed characteristic homogeneity and cobblestone-like morphology. Bars: 400 µm. (B) Representative images of capillary-like tubes formed on Matrigel by late EPCs. Bars: 400 µm. (C) Late EPCs were identified as double-positive for Dil-acLDL (red) uptake and lectin (green) binding affinity. Bars: 200 µm. (D) FACS analysis showing the immuno-phenotype of late EPCs using several endothelial cell-specific markers: vWF, CD31, VEGFR-2 and VE-cadherin. (TIF) [file pone.0067675.s004.tif]
