# Supplementary material for: Shear Stress Regulates Late EPC Differentiation via Mechanosensitive Molecule-Mediated Cytoskeletal Rearrangement
Source: PLoS One. 2013 Jul 2;8(7):e67675. doi: 10.1371/journal.pone.0067675 (PMC3699607; doi:10.1371/journal.pone.0067675)
Supplement: Table S1 — Primers used in Real-time RT-PCR. (DOC) [file pone.0067675.s005.doc]

Table S1

Primer sequences

| Gene | Sequences |
| --- | --- |
| GAPDH | F: GGCACAGTCAAGGCTGAGAATG |
|  | R: ATGGTGG TGAAGACGCCAGTA |
| vWF | F: GCGTGGCAGTGGTAGAGTA |
|  | R: GGAGATA GCGGGTGAAATA |
| CD31 | F: GACAGCCAAGGCAGATGCAC |
|  | R: ATTGGAT GGCTTGGCCTGAA |
